# Effect of recreational beach tennis on ambulatory blood pressure and physical fitness in hypertensive individuals (BAH study): rationale and study protocol

**DOI:** 10.1186/s12889-020-10117-5

**Published:** 2021-01-06

**Authors:** Rodrigo Ferrari, Leandro de Oliveira Carpes, Lucas Betti Domingues, Alexandre Jacobsen, Paula Frank, Nathalia Jung, Joarez Santini, Sandra C. Fuchs, The BAH Study Group

**Affiliations:** 1 Postgraduate Program in Cardiology, Universidade Federal do Rio Grande do Sul, Porto Alegre, RS Brazil; 2 Exercise Pathophysiology Laboratory, Universidade Federal do Rio Grande do Sul, Porto Alegre, RS Brazil; 3 Sports and Exercise Training Study Group, Hospital de Clínicas de Porto Alegre, Rua Ramiro Barcelos 2350, Porto Alegre, RS 90035-903 Brazil

**Keywords:** Sand sports, Exercise, Aging, Post-exercise hypotension, Hemodynamics, Functional exercise, Adherence

## Abstract

**Background:**

Different physical activities are widely recommended as non-pharmacological therapies to reduce blood pressure. However, the effectiveness of exercise programs is associated with its continuity and regularity, and the long-term adherence to traditional exercise interventions is often low. Recreational sports emerge as an alternative, being more captivating and able to retain individuals for longer periods. Besides, sport interventions have demonstrated improvements in physical fitness components that are associated with a lower incidence of hypertension. However, no studies have investigated the effects of recreational sports on 24 h ambulatory blood pressure. The aim of the present study is to evaluate the effect of beach tennis training on ambulatory blood pressure and physical fitness in individuals with hypertension.

**Methods:**

This study will be a randomized, single-blinded, two-arm, parallel, and superiority trial. Forty-two participants aged 35–65 years with previous diagnosis of hypertension will be randomized to 12 weeks of beach tennis training group (two sessions per week lasting 45–60 min) or a non-exercising control group. Ambulatory (primary outcome) and office blood pressures, cardiorespiratory fitness, muscle strength/power and quality of life will be assessed at baseline and after the intervention period.

**Discussion:**

Our conceptual hypothesis is that beach tennis training will reduce ambulatory blood pressure and improve fitness parameters in middle-aged individuals with hypertension. The results of this trial are expected to provide evidences of efficacy of recreational beach tennis practice on blood pressure management and to support sport recommendations for clinical scenario in higher risk populations.

**Trial registration:**

ClinicalTrials.gov, NCT03909321. Registered on April 10, 2019.

## Background

Hypertension is considered the most important modifiable risk factor for cardiovascular disease [1, 2] affecting nearly one-third of adults in Brazil [3] and worldwide [4] with increased prevalence throughout lifespan [5]. Although the usual treatment of hypertension is based on blood pressure lowering drugs, changes in lifestyle through physical exercise has emerged as an effective strategy for prevention and treatment of this condition [6]. A number of studies have shown that aerobic, resistance and combined training can reduce 24 h ambulatory blood pressure in hypertensive participants [7, 8].

The effectiveness of physical exercise programs seems to be directly associated with its continuity and regularity, and the long-term adherence to traditional exercise interventions (i.e., resistance and aerobic training) is often low [9]. One of the problems raised by individuals who do not adhere to exercise is the monotony and smaller motivation that those activities provide to their practitioners [10]. In this regard, recreational sports emerge as an alternative, being more captivating and able to retain individuals for longer periods and to sustain prolonged benefits to its practitioners [11, 12]. Promising results have been described with the use of recreational team sports on cardiovascular parameters [10, 13], including reduced BP in the office among participants with hypertension [14]. Although office blood pressure can be used to assess and diagnose hypertension, data assessing blood pressure through ambulatory blood pressure monitoring can provide additional information related to the benefits of recreational sports on blood pressure management, and future studies should investigate the effects of recreational sports on 24 h blood pressure.

The age-related decline in physical fitness components (i.e., cardiorespiratory fitness, muscular strength and power, balance, among others) [15] and physical activity levels [16] are also associated with increased prevalence of cardiovascular diseases. Different recreational sports, especially soccer [17] and other contact team sports [18, 19], have been studied over the last 10 years, suggesting that different sports could be effective for improving physical fitness and could be important for reducing risk of hypertension [20, 21]. In this regard, other sports modalities that can be practiced by middle-aged and older adults should be investigated to provide more information on this topic.

Although participation in recreational sports can be enjoyable, it may also face some difficulties to obtain high adherence, particularly due to musculoskeletal injuries [22]. Invasion sports, which usually demands a higher number of participants during the game, are prone to promote injuries [23]. Alternatively, sports with no physical contact and performed with few participants can be advantageous to improve participation [24]. In particular, beach tennis emerges as an interesting option based on easy accessibility, necessity of few participants to play, and lower risk of injury compared to traditional invasion sports [25]. Beach tennis is a racket sport played on a smaller sand court instead of the traditional tennis court, usually on the beach. It can be performed by different age groups and levels of conditioning/skills with only 2 to 4 participants per game. Nowadays, it is estimated that more than a million people practice the sport worldwide [25], and around sixty thousand practitioners in Brazil, a growth that represents an increase in the number of practitioners from 150 to 200% in the last year [26]. Recently, our research group evaluated the acute effects of a single session of beach tennis on different cardiovascular parameters in hypertensive individuals. During 45 min of beach tennis practice, the participants presented an average reserve heart rate (HR_reserve_) of 59–68% and there were no reported adverse events, confirming the potential of this sport to improve the cardiovascular profile in individuals with hypertension (Unpublished data; Trial registration: ClinicalTrial.gov n° NCT03909308).

To the best of our knowledge, no studies have investigated the chronic effects of team sports on 24 h ambulatory blood pressure and have assessed the effects of a beach tennis intervention in cardiovascular profile and physical fitness. Based on that, we designed this parallel randomized controlled trial to evaluate the effect of beach tennis training on 24 h ambulatory blood pressure and different physical fitness parameters in individuals with hypertension. The difference between the intervention arms in mean change from baseline in 24 h, daytime and nighttime systolic and diastolic ambulatory blood pressure at 12-weeks is the primary outcome; secondary outcomes are the difference between mean change in office blood pressure as well as cardiorespiratory fitness, muscular strength and power. We anticipate that 12 weeks of beach tennis training will reduce blood pressure when compared to a non-exercising control group. Additionally, our recreational beach tennis intervention will improve all physical fitness components in comparison to the baseline values.

## Methods/design

### Study design

This is a single-center, two-arms, parallel randomized controlled trial with concealed allocation, blinded measurers, with 12 weeks of follow-up analyzed using an intention-to-treat approach. The recruitment will take place between December 2020 and December 2021 through social media and banners. The protocol followed the recommendations for interventional trials (SPIRIT) 2013 guidelines [27].

### Study setting

The primary site of this study is the Center of Clinical Research at Hospital de Clínicas de Porto Alegre (Porto Alegre, RS, Brazil). The intervention center takes place at indoor and outdoor beach tennis courts (Porto Alegre, RS, Brazil), located outside to the hospital.

Inclusion criteria:

- Men and women (aged 35–65 years) with previous diagnosis of hypertension and using up to 3 of any class or type of anti-hypertensive drugs
- Not engaged in structured exercise programs in the 3 months prior to the start of the study
- Able to be enrolled, based on resting electrocardiogram (do not present arrhythmias, conduction disorders, coronary ischemic conditions or pathophysiological and structural abnormalities of the heart) and questionnaire about the participant’s clinical and osteoarticular symptoms
- Signed a written consent form for participation in the study

Exclusion criteria:

- Cardiovascular disease detected by acute myocardial infarction, angina, stroke or heart failure diagnosed in the last 24 months, as well as other chronic diseases (cancer, chronic heart failure with NYHA classes III or IV, kidney disease requiring dialysis, multiple sclerosis, Parkinson’s disease, among others)
- Current smokers or non-smokers for less than 6 months
- Body mass index over than 39.9 kg/m^2^
- Diabetic proliferative retinopathy

### Interventions

Participants will be randomly allocated to the beach tennis training intervention (BTT) or to the control group (Con), which will not be submitted to intervention and will be instructed not to engage in any kind of structured physical exercise training and to keep the life activities identified at baseline. The International Physical Activity Questionnaire (IPAQ) will be applied before and after the intervention in order to assess the level of physical activity.

#### Beach tennis training

In BTT, supervised beach tennis sessions will be performed two times per week. Each session will be composed of an initial period of 10 min of warm up and technical exercises (i.e., serve, volley, forehand and backhand). After that, 3 games of 10–15 min each (weeks 1–4: 3 × 10 min; weeks 5–8: 3 × 12 min; and weeks 9–12: 3 × 15 min) with an interval of 2 min between each game will be played in pairs (i.e., 2 versus 2). In case of less than 4 participants are able to play at the same time, the games can also be played individually (i.e., 1 *versus*1). As an objective measure of exercise intensity, heart rate (HR) will be continuously recorded with a Polar HR monitor (Polar FT7, Finland) during the beach tennis sessions, and data related to the average HR_reserve_ and maximal HR will be used to characterize the intensity throughout the sessions. We previously tested the physiological demand of beach tennis practice using the same methods and found an average HR of 59–68% HR_reserve_ during the beach tennis session lasting 45 min in participants with hypertension (Unpublished data; Trial registration: clinicaltrials.gov n° NCT03909308).

During the first week of beach tennis training, the participants will perform one or two familiarization sessions on the sand court in order to introduce basic beach tennis rules and game techniques. If necessary, one extra familiarization session will be allowed to those who need more practice to be able to play the game. Based on these familiarization sessions, two research team members with previous experience in beach tennis will classify each participant into three categories: beginner (poorly coordinated racket movements and little movement on the court), intermediate (more agile and coordinated movements), or advanced (broad dominance in all aspects of the game), in order to organize the schedule of beach tennis sessions by matching players of the same performance level. We believe that ensuring high motivation and adequate intensity during any sport practice is important and to organize the games using participants at a similar performance level will help to get it. *Control group -* In Con, participants will be advised to not change their daily usual activities and to not participate in any structured physical exercise program during this period. At the end of the study period after completing pre and post intervention assessments, we will offer 4 weeks of beach tennis practice to the participants of the control group.

### Outcome measurements

Outcomes will be assessed for all randomized participants before and after the intervention sessions, irrespective of attendance or completion status. For participants who drop-out of the study at any time after the randomization, researchers will use contact information to invite such participants to undergo the end-study outcome assessments (12 weeks after the beginning of intervention).

#### Primary outcome

The primary outcome is the change from baseline in 24 h, daytime and nighttime systolic and diastolic blood pressure measured by ambulatory blood pressure monitoring at 12 weeks.

#### Secondary outcomes

Secondary outcomes include the average change in office systolic and diastolic blood pressure as well as cardiorespiratory fitness, muscular strength and power and quality of life evaluated at baseline and after 12-week intervention in both arms. Additional measures of office blood pressure and enjoyment level during beach tennis sessions will be assessed at weeks 1, 4, 5, 8, 9 and 12.

### Participant timeline and data collection

**Fig. 1:**
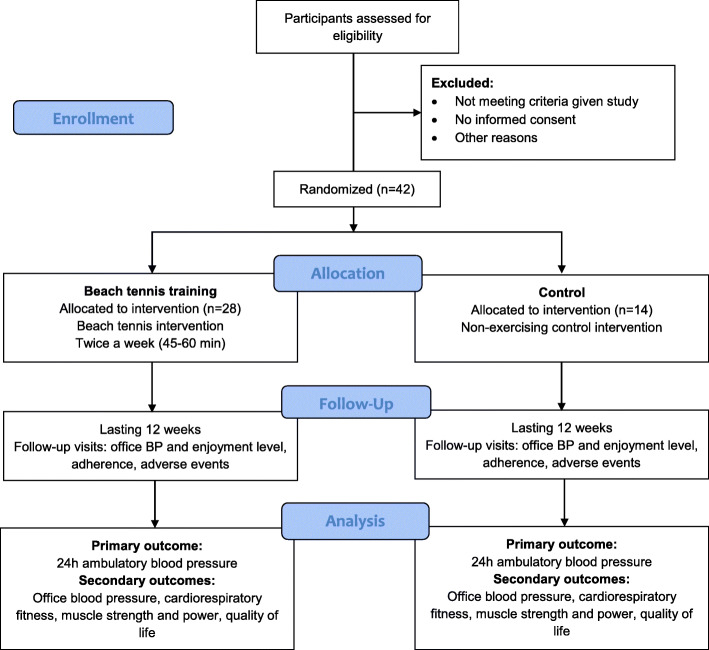
Recruitment and randomized allocation group from the CONSORT 2010 flow diagram

Participants will be recruited from electronic medical records, phone calls, e-flyers in social media, word of mouth, and personal references. Patients potentially eligible for the study will be contacted by telephone by the trial investigator, who will explain the study and ascertain the patient’s interest to participate. Those who accept to participate will attend the Center for Clinical Research of Hospital de Clínicas de Porto Alegre, where the study consultations will be performed, to sign a consent form. The schedule of enrollment, interventions, and assessments is presented in Table 1. The allocation of participants and timeline are described below and presented in Fig. 1.

**Table 1:**
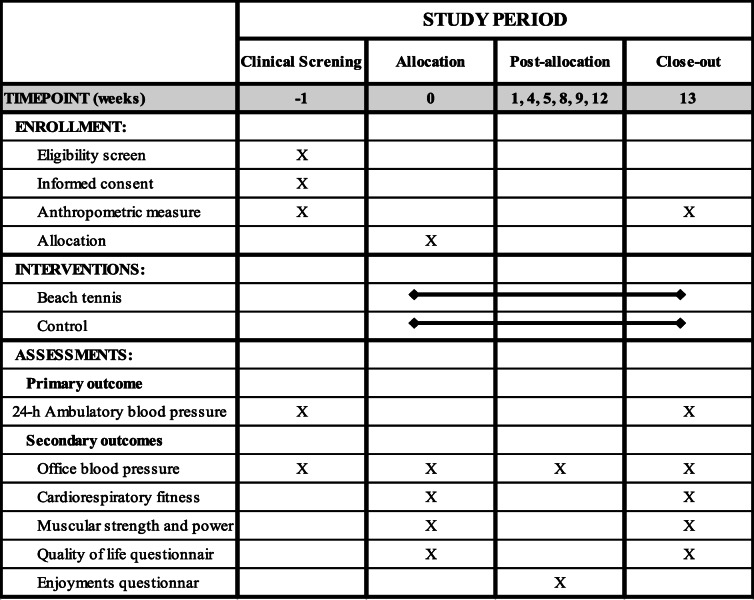
Schedule of enrollment, interventions, and assessments from SPIRIT guidelines

Those who signed the consent will undergo a clinical screening, resting electrocardiogram, anamnesis with sociodemographic, anthropometric and clinic data assessment, and questionnaire to evaluate quality of life. After that, the participants will perform a familiarization with strength and power muscle tests and with the mask that will be used in the cardiopulmonary test. In the second visit, participants will place the equipment of ambulatory blood pressure monitoring. On the day after, each participant will return at the laboratory to remove the equipment and perform evaluations of the cardiorespiratory fitness, muscular strength and power. All variables are assessed at baseline and at study completion. Participants will be asked to maintain their habitual activity and dietary habits at the time of recruitment.

### Measurement of primary outcome

*Ambulatory blood pressure* will be assessed throughout 24 h in intervals of 15-min in daytime and 20-min in nighttime periods. Daytime period starts at 7 AM and night-time starts at 11 PM. Participants will receive a formulary to note day activities, symptoms, sleep time and wake-up time. Each exam is considered valid when at least 14 measurements during the day and seven measurements at night [28]. If any exam is not considered valid, a new exam will be conduct up to 48 h between them. The participants will be instructed to avoid physical exercises and alcohol ingestion 24 h prior to exam. Ambulatory blood pressure will be assessed using an automatic oscillometric device (ABP 2400, Mortara, Milwaukee, EUA).

### Measurement of secondary outcomes

*Office blood pressure* will be measured after 10–20 min of rest, with the participant sitting quietly in a chair, according to standardized guidelines [29]. Three measurements, 1–2 min apart, are performed in the arm with the highest initial value. The average of the two measurements is considered. Furthermore, post-exercise hypotension will be measured during intervention period throughout the first session of weeks 1, 4, 5, 8, 9 and 12. In these sessions, blood pressure will be assessed at the beginning of session (after 10–20 min rest) and at the end of session in intervals of 15-min, during 60-min. The measures will be taken using a validated automatic oscillometric device (HBP-1100, OMRON Healthcare). The participants should avoid caffeine, exercise and smoking for 24 h before the measurements.

*Cardiorespiratory fitness* will be assessed using an incremental exercise test on a treadmill in order to determine peak oxygen consumption. The protocol will consist of an initial velocity of 3.5 km/h with 1% inclination for the first 2 min. Thereafter, velocity and grade will be incremented by 0.4–0.6 km/h and 0.5–1.0% inclination, respectively, every 1 min until the participants achieve their volitional exhaustion [30]. The expired gas will be analyzed using a metabolic cart (Metalyzer 3B Cortex, Leipzig, Germany). Blood pressure, electrocardiogram and heart rate will be continuously monitored and recorded throughout the test. The incremental exercise test will be conducted under the direct supervision of a licensed physician.

#### Muscular strength and power

*Countermovement jump test* will be assessed using My Jump2 software for mobile [31]. The participant will perform a warm up consisted of 10 squat strength exercise plus 3–5 jumps with 2-min interval. For the correct execution of the test, participant will be instructed to maintain orthostatic position keep their hands on their waist throughout the test, to perform the transition phase between the squat and flight phase quickly, to jump as fast as possible and to land at the same starting point. Three attempts will be performed with 30-s rest interval.

*Medicine ball throwing test* will be assessed using a medicine ball of 1 kg for women and 2 kg for men. Each participant will remain seated at the floor in an upright standing posture, with 90° abduction shoulder position, arms shoulder-aligned and flexed elbows. We will instruct to throw the ball as fast as possible, performing full-speed movement. Three attempts will be performed with a 1-min rest interval.

*Chair-stand test* will be conducted using a folding chair without arms, with a seat height of 43.2 cm. The test will begin with the participant seated in the middle of the chair, back straight, feet approximately shoulder-width apart and placed on the floor at an angle slightly back from the knees. Arms should be crossed at the wrists and held against the chest. At the signal “go”, the participant will stand up (body erect and straight) and then return to the initial seated position. The total number of stands executed correctly within 30-s and time to perform the first five repetitions will be used during the analyses [32].

*Isometric handgrip strength* will be measured in both arms with an analogic hand dynamometer (Jamar Sammons Preston Rolyan, Bolingbrook, IL, USA). The participant will remain seated with upright posture, placing the forearm parallel to the ground (elbow flexed at 90°). Thereafter, the participants will be instructed to perform a maximal squeezing contraction with sustained (isometric) effort lasting 5-s. Three attempts will be performed in each hand with 30-s rest intervals.

*Quality of life* will be evaluated through WHOQOL-BREF questionnaire [33]. The questionnaire contains 26 questions and is divided into four different domains (physical, psychological, social and environmental). The participant alone will answer the questionnaire and researchers provide help only if requested.

*Enjoyment* can define the level of fun in the different interventions and we will assess this variable through the Physical Activity Enjoyment Scale (PACES). The scale is composed of 18 items in a bipolar affirmations format (i.e., “amused” versus “not-amused”) punctuated in a range from the minimum value “1” to the maximum value “7” [34].

### Data management

Data will be collected on standardized paper forms identified by participant number and trial ID and containing instructions for standardized operational procedures. From these forms, we will proceed with double data entry for primary, secondary, and additional outcomes. All data will be stored and managed using the Research Electronic Data Capture (REDCap), an electronic data capture tool hosted at the Hospital de Clínicas de Porto Alegre.

### Sample size and power calculation

Sample size for the primary outcome was calculated using estimates of effect sizes from randomized clinical trial with similar design [14]. This estimation was set a priori based on a behavioral intervention for middle-aged patients with hypertension and analyzed in an intention-to-treat approach. A sample size of 42 individuals with hypertension on 2:1 ratio (BTT, *n*= 28) and (Con, *n*=14) allowing a dropout rate of 10%, will be able to detect a difference of 4 ± 6 mmHg in systolic 24 h blood pressure between groups. Statistical power was set at 90% including a type I error rate of 5%. WinPepi software calculator was used to estimate the sample size [35].

### Randomization and allocation concealment

The randomization list will be generated by an epidemiologist using web software (www.random.org), with blocks of random sizes that will not be disclosed to ensure concealment. Groups will be generated by stratified randomization per age (35–50 and 51–65 years) and baseline systolic ambulatory blood pressure in order to provide homogeneity on these prognostic factors. The epidemiologist will not participate in the recruitment or assignment to intervention sessions. The outcome assessors and data statistical analyses will be double masking to the endpoints of study. In contrast, participants and the beach tennis instructors will be blinded to the randomization list until the moment of assignment.

### Strategies of study retention

During study period, we will use phone calls/ text messages to inquire for any adverse events or if a participant misses a beach tennis session reschedule missed sessions. The phone calls schedule will be ceased for participants declaring their withdrawal from the study. Thereafter, we will make available to participants three non-consecutive days in different time and turn to perform the interventions.

Measures of adherence to beach tennis intervention will be reported as group averages and operationalized as attendance and compliance. Attendance will be monitored through session’s frequency recording and will be treated as the percent of beach tennis intervention sessions experienced by a participant given the total number of scheduled sessions. Adherence will be treated as the percent of intervention sessions fully accomplished without protocol deviations given the total number of scheduled sessions.

### Safety assessments

Data on all adverse events will be collected and included in medical reports, which will be forwarded to the health authorities. To ensure patient safety, we will monitor any adverse events occurred during the study, and all participants will be monitored throughout the study follow up and during 4 weeks after study finished.

### Statistical analysis

The endpoints will be analyzed using a full analysis set including all randomized participants, therefore allowing intention-to-treat analyses. A second set of per-protocol analysis will be performed including participants that completed the trial with adherence to at least 80% of the beach tennis intervention sessions (≥ 19 sessions for participants allocated in the BTT program).

Data distribution will be analyzed using Shapiro-wilk test with analysis of histogram and Q-Q plots in combination. Data will be expressed as means and standard error for variables with normal distribution or medians and interquartile range for non-normal distributions and 95% confidence intervals (95%CI). Ambulatory blood pressure will be analyzed as daytime, nighttime and 24 h systolic and diastolic blood pressure. Primary and secondary outcomes will be analyzed using generalized estimating equation model (GEE) for correlated measures. Adjustment for multiple comparisons will be accomplished using Sequential Bonferroni test. All analysis will be conducted using SPSS Statistics for Windows, version 18.0 (IBM corp., Armonk, NY, USA).

### Dissemination policy

We intend to disseminate the methods and findings of the BAH study through individual explanation about the main findings and practical application of the study to each participant; press releases written by journalists and using our personal social media to the general public; and submit scientific manuscripts related to our main findings.

## Discussion

Although several types, formats, and frequencies of physical activity interventions are available to reduce blood pressure, the optimal exercise training remains controversial. Recreational sports can be effective to reduce blood pressure [10, 21] and an enjoyable alternative to increase adherence [36], but the effects of recreational sports on 24 h ambulatory blood pressure are lacking. Even though the beneficial adaptations on different physical fitness parameters following team-sport training have been established, access to suitable training facilities is often limited. This is the first study to evaluate the effects of team sport on 24 h ambulatory blood pressure among hypertensive participants. Moreover, our study aims to confirm the potential benefits of a beach tennis intervention in different physical fitness parameters. Based on easy access, the need for few participants to play, and less risk of injury compared to traditional contact sports [22, 35], beach tennis seems to be an advantageous option.

Team sports interventions have also demonstrated improvements in different physical fitness components that are comparable to traditional exercise programs [13]. The increase in cardiorespiratory fitness and muscular strength improves the ability to perform daily activities better [36, 37]. Besides, cardiorespiratory fitness and muscular strength levels are inverse and independently associated with cardiovascular disease and death from all causes [37, 38]. Moreover, maintaining or improving cardiorespiratory fitness in adulthood is associated with a lower incidence of hypertension [40]. Together, exercise strategies that simultaneously reduce blood pressure and increase physical fitness should be emphasized in individuals with hypertension. This study will provide information on the effect of a promising and easy to play sport on 24 h blood pressure in individuals with hypertension. Recreational beach tennis has the potential to be used as a retention and continuity strategy, promoting a broad-spectrum of health improvements associated with pleasure and satisfaction during physical activity.

## Data Availability

Not applicable.

## Abbreviations

HR: Heart Rate
IPAQ: International Physical Activity Questionnaire
PACES: Physical Activity Enjoyment Scale
BTT: Beach tennis intervention group
Con: Non-exercising control group
REDCap: Research Electronic Data Capture
95%CI: 95% Confidence Intervals
GEE: Generalized Estimating Equation model
